# Maryland State Dental Society

**Published:** 1868-03

**Authors:** S. H. Williams, S. M. Field

**Affiliations:** Pres.; Rec. Sec.


### CORRESPONDENCE.

*"Maryland State Dental Society."*

THIS association held its regular monthly meeting in Baltimore, Jan. 30th, at the office of Dr. J. B. Bean.

The subject of discussion was "treatment of the dental pulp preparatory to filling."

Dr. Volck, who had been appointed by the society, at the December meeting, to make an analysis of "Welsh's nerve paste," reported that he had done so, and found it to contain arsenic, and other substances which he supposed to be morphia and a small quantity of rose matter. Dr. V's demonstration by means of Marsh's test, of the presence of arsenious acid in the preparation referred to, was very beautiful.

Dr. Bean explained, in very full detail, his method of employing aluminum as a base for artificial teeth; he exhibited his apparatus for the purpose, and explained minutely, all the steps of the process.

Dr. Arthur offered the following propositions to the association—

1.—That caries will attack the proximate surfaces of all the teeth, except the inferior incisors, of the great majority of persons in the United States, at the present day.

When caries of the superior incisors occurs on the proximate surfaces previously to the twelfth year, its occurrence, sooner or later, on the same surfaces of all the teeth, except the inferior incisors is almost certain. In the greater number of such cases the caries will show itself before the twentieth year.

This predisposition to dental caries is greater in the female sex.

2nd.—That caries is not liable to occur, at the points indicated, unless the teeth are in contact.

3rd.—That an artificial, permanent, separation of the teeth will arrest superficial caries or prevent its occurrence if the attack has not actually begun.

4th.—That it is a popular fallacy to suppose that caries necessarily follows the removal of the enamel.

5th.—That the most efficient means of preserving the teeth is to anticipate the attack of caries by separating them, when it is ascertained that caries is likely to occur on the proximate surfaces.

Dr. Arthur submitted the above propositions to the society, as the result of more than twenty-five years careful observation of the phenomena of dental caries, and stated his readiness to defend them against any one who felt disposed to dispute his conclusions.

Dr. Volck declared his intention of controverting the propositions of Dr. A. but desired some further time for preparation.

Dr. A. stated his readiness to meet him in discussion of the subject at any time.

S. H. Williams, *Pres.*

S. M. Field, *Rec. Sec.*

---

